# Analysis and Prediction of Wear Resistance on Grind-Hardening Layer Considering Different Friction Conditions

**DOI:** 10.3390/ma18050975

**Published:** 2025-02-21

**Authors:** Yu Guo, Minghe Liu, Yiming Zhang

**Affiliations:** 1 School of Mechanical Engineering and Automation, Northeastern University, Shenyang 110819, China; guoyu@me.neu.edu.cn; 2 School of Mechanical Engineering, Shenyang Jianzhu University, Shenyang 110168, China; zhangyiming@htgd.com.cn

**Keywords:** grind-hardening, hardened layer, wear resistance, friction condition

## Abstract

The grind-hardening process is capable of generating a martensitic-based hardened layer on the workpiece surface. The production of a hardened layer can significantly improve the application properties of the workpiece. In fact, theoretical research on the wear process of hardened layers is a powerful key to promoting the grind-hardening process, which is the main focus of the current experimental study. For this purpose, the paper carries out the grind-hardening experiment on AISI 1045 steel first by discovering the formation mechanism of the hardened layer. Then, friction and wear experiments are conducted on hardened workpieces to analyze the influence laws of different conditions on the friction coefficient and wear morphology, as well as its profile. On this basis, combined with the Archard wear model, finite element simulations are carried out on the wear process with different friction conditions. The wear depth is effectively predicted. The results show that the wear depth gradually rises with the increase in friction load and frequency. Additionally, considering different friction conditions, the errors between the predictive and experimental values of the wear depth with both average friction coefficient and variable friction coefficient are 4.36–15.22% and 1.57–10.4%, respectively, which validates theoretical research on the wear resistance of the hardened workpiece.

## 1. Introduction

The grinding process, as a precision machining approach, plays an irreplaceable role in achieving the parts with high precision and low roughness surfaces during the mechanical manufacturing [1]. Actually, the process is essentially a micro-cutting process, which removes the material on the workpiece in a minimal amount to obtain accurate dimensions and high-quality surface by the abrasives densely distributed on the wheel [2,3]. However, the grinding process also faces many challenges and problems in practical applications, although it is currently the most commonly used final machining method for workpieces. From the perspective of machining efficiency, to ensure the surface integrity of the workpiece, the material removal rate per unit time is relatively low because of the small size of the abrasives on the wheel, whose processing efficiency is lower than other methods [4]. Besides, the severe plastic deformation and the friction between the abrasives and the workpiece generate a large amount of heat during the process. If the heat cannot be dissipated in a timely and effective manner, it will lead to a series of problems, such as burn on the workpiece surface and an increase in residual tensile stress. These problems may further affect the final application performance. Although the use of a large amount of coolant is helpful to reduce the grinding heat, it will not only increase the processing cost but also cause environmental pollution. Therefore, the development of new grinding processing technologies has always been a research hotspot in the field of mechanical manufacturing and conforms to the needs of modern industry as well.

The grind-hardening process, as an emerging integrated manufacturing technology that combines the grinding process and surface quenching theories [5], mainly utilizes the heat generated instantaneously in the processing area to conduct quenching treatment on the surface of non-hardened steel workpiece directly. Within a short period of time, it makes the austenite on workpiece surface transform into martensite, thereby achieving the combination of surface quenching treatment and high efficiency grinding process, as well as improving the application performance (such as wear resistance, fatigue resistance and corrosion resistance, etc.) of the workpiece surface [6]. Given the advantages of the grind-hardening process, scholars have significantly promoted the development of this process by extensive research in recent years. Brinksmeire and Brockhoff et al. [7,8] found, through experimental research, that a hardened layer formed on the workpiece surface due to microstructure transformation in the grind-hardening process. Meanwhile, both the hardness and wear resistance were higher than those of non-hardened workpieces. Zarudi et al. [9] conducted grind-hardening experiments on annealed 42CrMo steel. The results showed that the hardened penetration depth of the workpiece could reach 0.6 mm, and both the fatigue resistance and wear resistance were significantly enhanced. Wang et al. [10] carried out grind-hardening experiments on 40Cr steel and analyzed the formation mechanism of the hardened layer in reciprocating grinding by comparing the microstructures and properties of the hardened layers in single-pass and reciprocating grinding. Nguyen et al. [11] designed grind-hardening experiments using liquid nitrogen as a coolant. The research found that the cooling method was beneficial to make a hardened layer with excellent surface quality. Meanwhile, it had the effect of reducing the surface residual stress and without surface oxidation phenomenon. Liu et al. [12] adopted the finite element method (FEM) to simulate the temperature distribution on workpiece surface and its instantaneous changes during the grind-hardening process and effectively predicted the hardness of the hardened layer. Salonitis [13] applied the cellular automata (CA) method to predict the evolution process of the microstructure of the hardened layer innovatively and achieved excellent results. Deng et al. [14] simulated the transformation process of austenite nucleation, growth, and coarsening during the formation of the hardened layer and predicted the hardness and residual stress. Ehle et al. [15] discovered the formation mechanism of the hardened layer and the influence of the inhomogeneous matrix material on the hardened layer by analyzing the correlation between the microstructure transformation on the workpiece surface and the internal load. Huang et al. [16] analyzed the variation laws of the grinding force during the grind-hardening process and put forward a simulation model for predicting the distribution of residual stress based on the variable grinding force. This process was proved to generate residual compressive stress on the workpiece surface. Guo et al. [17] simulated the microstructure evolution process of the hardened layer by combining FEM with CA, and effectively predicted the hardness with different grinding parameters. Shi et al. [18] verified that the formation of the hardened layer in the grind-hardening process significantly enhanced the corrosion resistance of the workpiece through experiments. Based on the prediction of the austenite-martensite transformation, an innovative prediction model for hardened penetration depth was established and verified through grind-hardening experiments by Lerra et al. [19]. Mao et al. [20] determined the critical grinding depth for the formation of the hardened layer on the basis of analyzing the microstructure and physical properties with different grinding parameters.

From the above literature, numerous scholars adopted the method of physical experiments to prove the feasibility of the grind-hardening process in realizing surface strengthening in the early stage of research. Subsequently, FEM and CA were applied to explore the grinding temperature distribution on the workpiece surface and the microstructure transformation for revealing the formation mechanism of the hardened layer. Although the research on the grind-hardening process currently has a certain theoretical basis, the relatively short time has led to the fact that the research on resistance of the wear, fatigue, and corrosion of the hardened layer is still at the experimental stage [7,9,18]. The insufficiency of theoretical research in this aspect has resulted in the inability to design the grinding parameters according to the requirements of the application performance on the workpiece surface and has hindered the usage of the process in actual engineering, whereas wear is one of the main causes leading to the failure of parts. According to statistics, more than 80% of the failures of mechanical parts can be attributed to wear at different levels [21]. Consequently, taking the wear resistance of the hardened layer as the research object, this paper studies the wear process of the hardened layer with different friction conditions basing on grind-hardening experiments and friction and wear experiments, together with the Archard wear model and FEM. Besides, the wear depth is effectively predicted. The research provides a parametric basis for improving the theoretical framework of the grind-hardening process and develops an optimized design approach for the grinding parameters that meet the requirements of wear resistance with different working conditions.

## 2. Grind-Hardening Experiment

### 2.1. Experimental Conditions

First of all, the grind-hardening experiment should be carried out ahead of the research on the wear resistance of the hardened layer, which is conducted on the BLOHM Orbit 36 CNC precision surface grinder (BLOHM, Hamburg, Germany). The machining material is non-quenched AISI 1045 steel. The material dimensions are 35 mm (length) × 25 mm (width) × 25 mm (height). A 300 mm (diameter) × 30 mm (width) alumina wheel, whose grain size is F46, is adopted in the experiment. Meanwhile, dry grinding is capable of ensuring that the workpiece surface reaches the transformation temperature. As can be seen from many previous studies [6,12], grinding parameters are the key factors influencing the formation of the hardened layer. Meanwhile, the reasonable selection of grinding parameters can ensure that the hardened layer has outstanding application performance. Therefore, in order to obtain a hardened layer with excellent performance, considering the comprehensive influence of grinding parameters on the depth, hardness, and surface roughness, as well as the wear resistance with different friction conditions in subsequent research, only one set of parameters is designed for the grind-hardening experiment, with a grinding depth of *a*_p_ = 0.2 μm, a feed rate of *v*_w_ = 0.02 m/s, and a wheel speed of *v*_s_ = 35 m/s.

### 2.2. Microstructure

**Figure 1:**
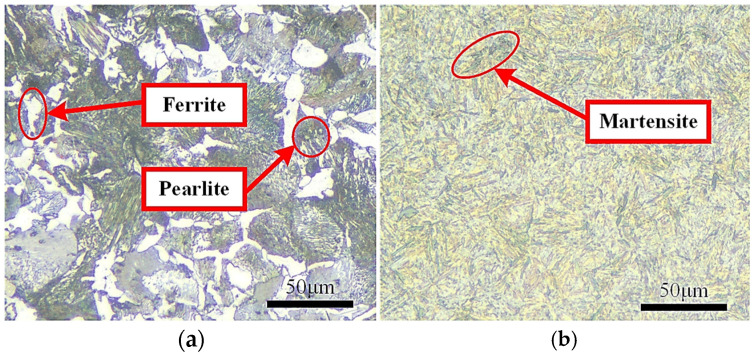
Microstructure of workpiece surface: (**a**) Matrix; (**b**) Hardened layer.

The workpiece surfaces both before and after grind-hardening are ground, polished, and corroded, and their microstructures are observed through a metallographic microscope, which is shown in Figure 1. From Figure 1a, the workpiece matrix is mainly composed of proeutectoid ferrite and pearlite. After grinding, a large number of lath martensite appears on the surface, as shown in Figure 1b. Combined with the characteristics of the grind-hardening process and the metal phase transformation theory, the formation of martensite is mainly due to the fact that a large amount of grinding heat is generated in the contact area between the wheel and the workpiece, which causes the austenite transformation of the matrix on workpiece surface during the grind-hardening process. As the wheel moves, the cooling rate and the supercooling degree make the austenite undergo a lattice rearrangement from γ-Fe to α-Fe, without diffusion of iron and carbon atoms. Eventually, a supersaturated interstitial solid solution-martensite in α-Fe of carbon is formed on the workpiece surface.

### 2.3. Microstructure Distribution Along the Cross-Section

**Figure 2:**
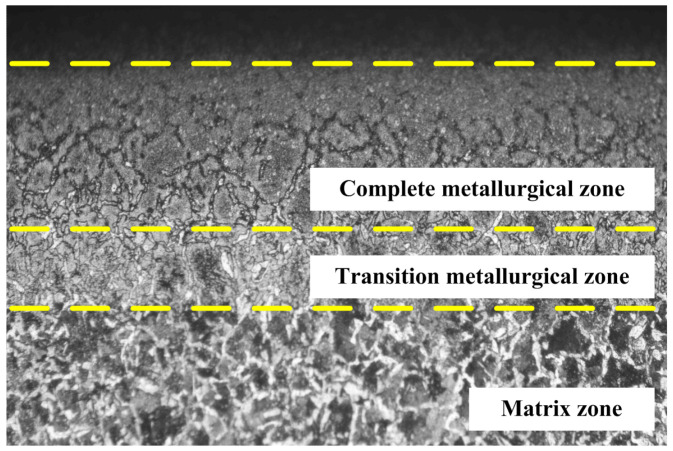
Microstructure distribution of the cross-section.

Figure 2 shows the microstructure distribution along the cross-section of the workpiece. It can be noticed that there are apparent differences in microstructure distribution as the distance from the surface increases in the figure. This is mainly because an extremely high grinding temperature on the surface transfers to the interior of the workpiece by means of heat conduction, which forms a non-uniform temperature field. When the surface exceeds Ac3 temperature, the initial microstructure in this area is completely austenitized first. Then, a complete metallurgical zone dominated by martensite is formed after rapid cooling. Since the temperature gradually decreases inside the workpiece during the conduction process, partial austenite transformation occurs in the subsurface within the temperature range from Ac1 to Ac3. The final microstructure is composed of both fine martensite and ferrite, which is usually referred to as the transition metallurgical zone. Meanwhile, the microstructure is unchanged in the matrix since the temperature is lower than Ac1, which is still composed of proeutectoid ferrite and pearlite.

## 3. Friction and Wear Experiment

### 3.1. Experimental Conditions

Reciprocating sliding friction and wear experiment is carried out on the hardened layer using a multifunctional friction and wear tester, with dry friction as the lubrication condition. The friction pair is AISI 1045 steel after grind-hardening and silicon nitride (Si_3_N_4_) ball with a diameter of 6.35 mm, respectively. For better analyzing the influence mechanism of friction conditions on the wear resistance of the hardened layer, it is necessary to fully consider the differences in the friction coefficient, wear surface morphology, and wear depth and width with different friction conditions when selecting friction parameters. Therefore, an experimental scheme with different loads and frequencies is designed. The specific scheme is shown in Table 1.

### 3.2. Friction Coefficient

**Figure 3:**
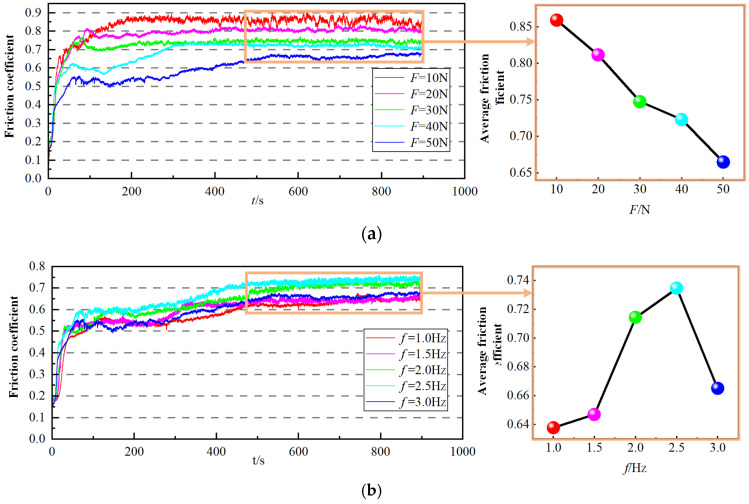
Friction coefficient with different friction conditions: (**a**) *f* = 3.0 Hz; (**b**) *F* = 50 N.

Figure 3 depicts the time-varying curves of the friction coefficient of the hardened layer with different conditions. Obviously, the friction coefficients in different loads and frequencies show a trend of increasing first and then leveling off in the figures. This is due to the presence of a third body medium with lubricating effects on the workpiece surface at the initial contact, such as oxide films and adsorption films, and the friction coefficient is relatively small during the rapid rising stage. When the friction coefficient increases to a certain range, a hardened effect appears on workpiece surface. Meanwhile, the friction coefficient will rise slowly as the spalled wear debris participates in the friction. As the experiment continues, the frictional behavior reaches an equilibrium state, which eventually makes the friction coefficient remain basically stable.

From Figure 3a, the variation of the friction coefficient of the hardened layer with different loads decreases gradually as the load grows. When the normal load increases, the shear stress on the hardened layer keeps rising, and the plastic deformation induced by friction also increases gradually. Moreover, the plastic deformation leads to a hardening effect on the worn surface and subsurface, further strengthening the friction surface. In addition, a large amount of friction heat generated during the dry friction process causes an oxide film to form on the hardened surface, which is also conducive to the reduction of the friction coefficient. It can be seen from Figure 3b that the variation of the friction coefficient is that as the frequency increases, the friction coefficient rises first and then drops. The reason is that the friction frequency mainly affects the friction coefficient through heat. Specifically, when the frequency increases, the friction distance within a unit of time grows as well. As a result, more friction resistance needs to be overcome, causing the friction coefficient to increase to some extent. However, when the frequency continues to increase until it exceeds a critical value, the dissipation of friction heat begins to decline, resulting in an accelerated oxidation rate on the worn surface. Meanwhile, the wear debris is more likely to be compacted into the surface, thus forming a protective layer and eventually causing the friction coefficient to decrease to some extent.

### 3.3. Worn Surface Morphology

**Figure 4:**
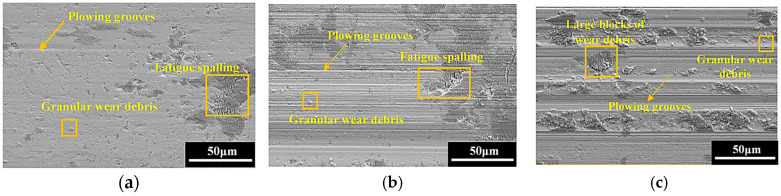
The worn surface morphologies with different friction loads when *f* = 3.0 Hz: (**a**) *F* = 10 N; (**b**) *F* = 30 N; (**c**) *F* = 50 N.

**Figure 5:**
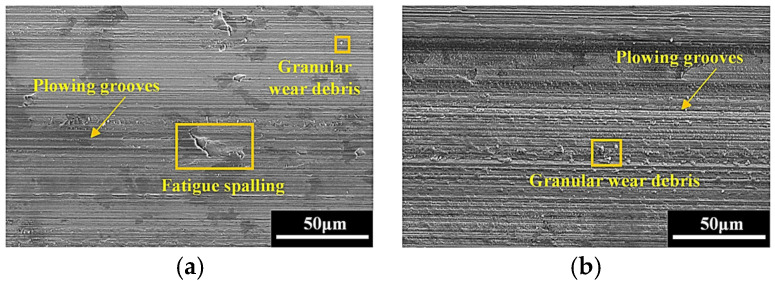
The worn surface morphologies with different friction frequencies when *F* = 50 N: (**a**) *f* = 1.0 Hz; (**b**) *f* = 2.0 Hz.

To better understand the wear behavior of the hardened layer, the field emission scanning electron microscope is adopted to characterize the microscopic morphology of the worn surface. From Figure 4, when the friction load is 10 N, the worn surface exhibits relative smooth feature, but sparse plowing grooves, tiny fatigue spalling, and a small amount of adhesive wear debris can still be observed. As the friction load continuously increased from 30 N to 50 N, the characteristics of abrasive wear and fatigue wear become more pronounced. In addition, the volume of the wear debris gradually expands, and an obvious adhesion occurs. Combined with Figure 5 and Figure 4c, it can be seen that as the frequency goes on gradually, the plowing on the worn surface intensifies progressively, and all the quantity, width, and depth of the plowing grooves increase as well. Moreover, large blocks of wear debris begin to be severely squeezed on the worn surface. This is mainly due to the rise in the frequency, the number of frictions, and the distance within a unit of time increase, making the wear speed faster.

### 3.4. Wear Measurement

**Figure 6:**
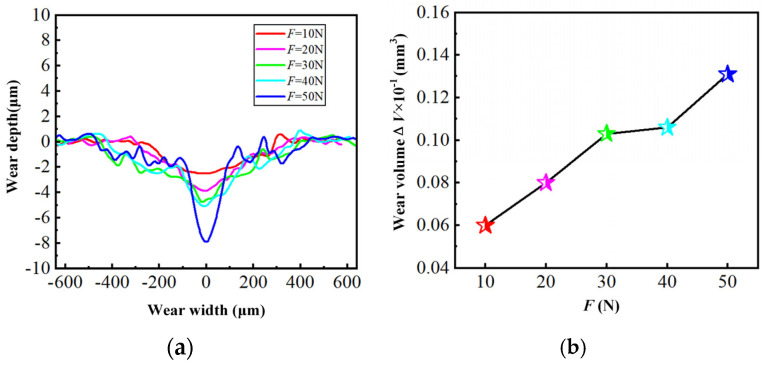
The wear cross-section and wear volume of the hardened layer with different friction loads: (**a**) Wear cross-section; (**b**) Wear volume.

**Figure 7:**
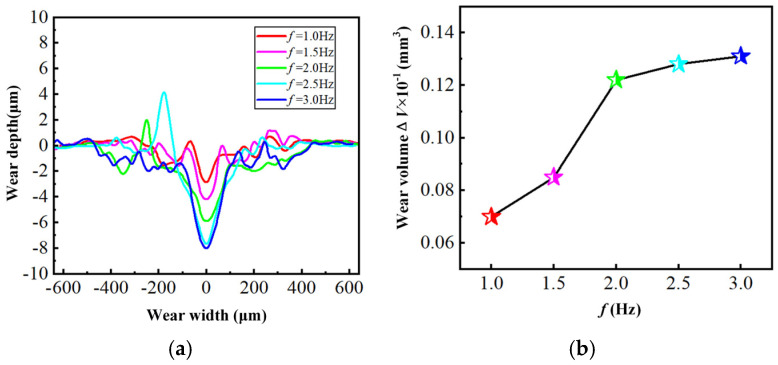
The wear cross-section and wear volume of the hardened layer with different friction frequencies: (**a**) Wear cross-section; (**b**) Wear volume.

The laser scanning confocal microscope is employed to detect the three-dimensional morphology of the worn surface on the hardened layer. Besides, the surface data and the wear volume are measured, applying the test software (OLS 4100). Figure 6 shows that the worn surface is U-shaped and accompanied by a material protrusion when the load is 10 N. Then, the worn surface gradually changes to a V-shape in the middle, while the wear depth on both sides is relatively small as the load continues to grow. It can also be seen from Figure 6 that both the depth and the volume of wear improves, and the friction load increases gradually. The rise of the load advances the shear stress and surface temperature, leading to the softening of the material on the worn surface, thus resulting in an increase in the amount of wear. From Figure 7, as the friction frequency increases, the depth and volume of wear also grows step by step because the higher frequency improves the number of frictions within the same period of time. In such circumstances, the heat generated by friction accumulates more on the surface, thereby causing the hardened layer to soften and eventually intensifying the wear. Both the load and the frequency are the key factors influencing the wear resistance of the hardened layer as seen in Figure 6 and Figure 7. Meanwhile, the increase in both leads to the acceleration of the wear rate.

## 4. Simulation of Friction and Wear

### 4.1. Methodology

Currently, the Archard wear model is widely used in the mechanical field [22], which describes sliding wear based on asperity contact theory [23]. According to the model, the material wear volume under a given sliding distance is directly related to the friction load, wear coefficient, and hardness of the softer material [24,25]. Therefore, the general form of the Archard wear model is described as:

(1) $$\frac{\Delta V}{S}=K\frac{F}{Hv}$$

where ∆*V* is the volume of wear; *S* is sliding distance on the surface; *F* is friction load; and *Hv* is the hardness of the softer material.

With the aim of analyzing the evolution process of the wear profile in the friction cycles, it is necessary to accurately calculate the wear depth at different positions of the surface. Suppose that the contact area is *A* at a certain moment and both sides of Equation (1) are divided by *A*, respectively. At present, the Archard wear model can be expressed as:

(2) $$\Delta h=K\frac{PS}{Hv}$$

where ∆*h* is the wear depth and Δ*h* = Δ*V*/*A*; *P* is the normal contact pressure and *P* = *F*/*A*.

Therefore, the relationship between the local increments of wear depth and sliding distance is achieved by the following equation:

(3) $$\frac{dh}{ds}=kP$$

where d*h* is increment of wear depth; d*s* is increment of sliding distance; *k* is the local wear coefficient obtained from friction experiments; and *k* = *K*/*Hv*.

### 4.2. Finite Element Simulation

#### 4.2.1. Finite Element Model

**Figure 8:**
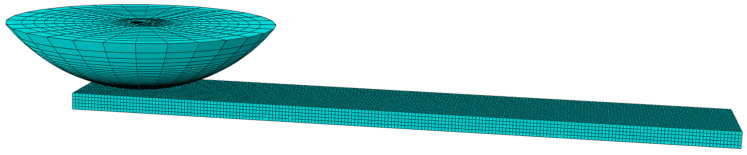
The finite element modelling and meshing of friction pair.

The grind-hardening workpiece and the Si_3_N_4_ ball are modelled with finite element software, whose material properties are listed in Table 2. Since only the contact area of the friction pair participates in the calculation, a local finite element model of the ball-block friction pair is established on the premise of ensuring the accuracy of the calculation and improving the convergence and computational efficiency. All components in the model adopt an 8-node linear brick element (C3D8R), whose meshing size of the model is 0.08 mm. Among them, the hardened workpiece is divided into 42,210 nodes and 34,000 elements, and the Si_3_N_4_ ball has a total of 17,335 nodes and 16,772 elements. The finite element modelling and meshing are shown in Figure 8.

#### 4.2.2. Boundary Conditions and Loading

**Figure 9:**
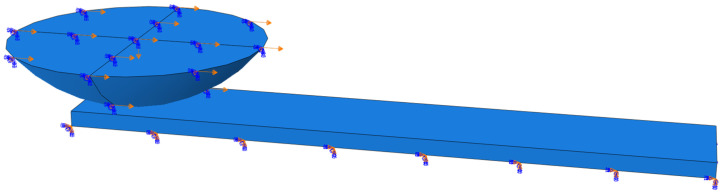
Schematic diagram of boundary conditions and loading of the friction pair.

According to the friction and wear experimental scheme, load and boundary conditions are set for the three-dimensional finite element models of the hardened workpiece and Si_3_N_4_ ball. The contact surface interaction is defined by the contact pair method, whose contact constraints are applied with the master-slave algorithm. Considering the stiffness difference, the bottom of the Si_3_N_4_ ball is designated as the master surface, and the workpiece surface is assigned as the slave surface. The normal behavior of the contact surface is defined as hard contact. The tangential behavior adopts Coulomb’s friction law. The contact problem is solved by the Lagrange multiplier. As can be seen from Figure 3, the friction coefficient of the hardened layer with different friction conditions changes non-linearly with time. Since the friction coefficient is a key indicator for evaluating the wear resistance of the material, it is necessary to express the variation laws of the friction coefficient with different friction conditions as functions. These functions are then applied in the contact conditions of the friction pair to simulate the wear process of the hardened workpiece more realistically. A normal load is applied at the middle node on the upper surface of the sphere model. The specific setting scheme is shown in Figure 9. Since the workpiece is fixed, it is necessary to fully constrain the bulk model (U1 = U2 = U3 = UR1 = UR2 = UR3 = 0). When the Si_3_N_4_ ball slides reciprocally along the *X*-axis direction relative to the workpiece, its model is thus constrained in the *Y*-axis direction and the three rotation directions (U2 = UR1 = UR2 = UR3 = 0). Besides, the displacement boundary conditions in the *X*-axis direction are set in tabular form to achieve the sliding motion consistent with the friction experiment. During the simulation, a dynamic explicit analysis step is adopted to simulate the friction conditions, and the duration of the analysis step is set to 0.01 s. It should be noted that, since finite element software cannot directly establish a wear mechanism model, the Archard wear model needs to be compiled by Fortran language and written into the UMESHMOTION subroutine during the simulation. Among them, the wear coefficient *k* in the model can be obtained through friction and wear experiments. It is assumed that the wear coefficient is the same at all positions in the entire contact area. During the solution process, the contact pressure and its distribution state of each node in the contact area are obtained by reading the finite element results of the friction pair contact simulation at different moments. Furthermore, according to the Archard wear model, the wear depth of each node at a certain moment can be calculated. On this basis, combined with the ALE (Arbitrary Lagrangian Eulerian) adaptive meshing technique, the displacement of nodes within the adaptive meshing area is adjusted according to the calculated results of wear depth. At this time, due to the application of friction load, real-time contact between the workpiece and the Si_3_N_4_ ball is always ensured. Subsequently, the above process is iterated, and the simulation of the wear process of the hardened layer is achieved eventually. Meanwhile, the displacement of each node in the contact area at different moments throughout the process is summed up to obtain the total wear depth [26,27].

#### 4.2.3. Discussion

(1) The influence of friction load on the wear resistance of the hardened layer

**Figure 10:**
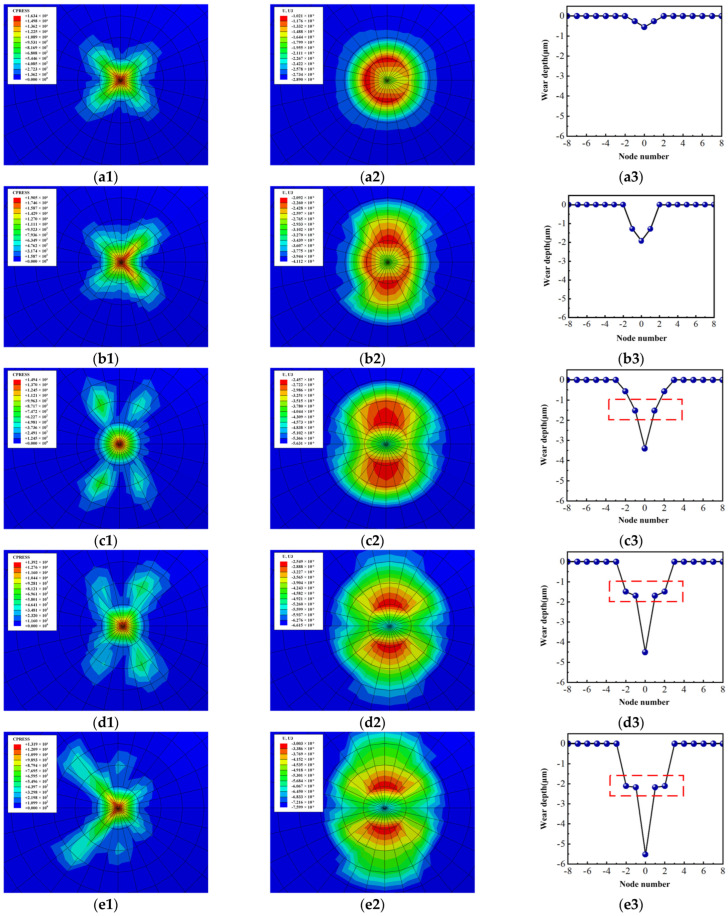
Wear process of the friction pair at different times when *F* = 30 N: (**a1**–**a3**), *t* = 66 s; (**b1**–**b3**), *t* = 266 s; (**c1**–**c3**), *t* = 466 s; (**d1**–**d3**), *t* = 666 s; (**e1**–**e3**),*t* = 866 s. (**a1**–**e1**), contact pressure of Si_3_N_4_ ball; (**a2**–**e2**), wear distribution of Si_3_N_4_ ball; (**a3**–**e3**), wear profile of hardened workpiece.

Figure 10 shows the contact pressure and wear distribution of the Si_3_N_4_ ball and the wear profile of the workpiece with the condition of *F* = 30 N at different moments. At the initial stage, the contact pressure is concentrated at the center of the friction area because the contact in the friction pair is approximately pointing contact, as seen in Figure 10(a1–c1). At this time, the contact area of the Si_3_N_4_ ball is close to a circular shape. Both the depth and width of wear on the workpiece are relatively small. When *t* = 266 s, the maximum contact stress between the friction pairs is still at the center of the friction area, whereas the stress has decreased due to the gradual increase in the contact area, whose contact form presents as an elliptical surface contact. By now, the maximum wear of the Si_3_N_4_ ball expands towards the surroundings along with the movement of the contact stress, resulting in a simultaneous increase in the depth and width of the wear on the workpiece (as shown in Figure 10(a2–c2). When the moment reaches *t* = 466 s, 666 s, and 866 s, respectively, the variation laws of the contact stress and contact area between the friction pairs are the same as those in the previous load steps. That is, as the friction proceeds, the depth and width of wear continue to expand due to the increase in the influence range and area of the contact stress (as shown in Figure 10(a3–e3)). However, it should be noted that the particular positions in the wear width direction showed smooth, inclined, and straight grooves at the above moments. This is mainly because the relatively large contact stress moves towards the edge of the contact area along with the increase in the contact area of the friction pair. Therefore, the stress at the edge of the contact area is greater than that inside due to the stress concentration. Meanwhile, it also leads to the accelerated wear of the edge materials. On this account, the large contact stress has a cyclic movement between the contact edge and the interior during the friction process and finally results in different wear shapes along the width direction of the profile at different moments.

**Figure 11:**
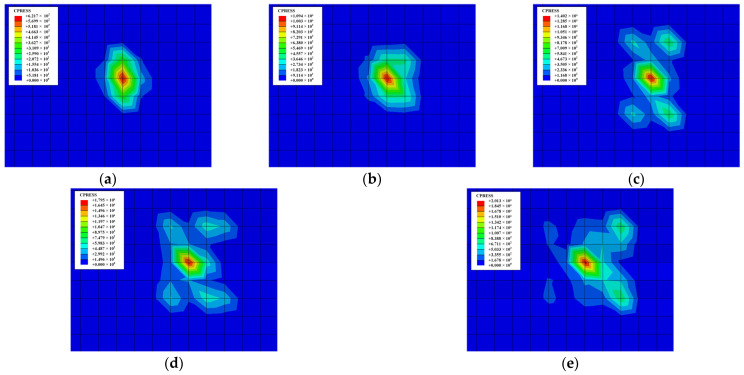
Contact stress distribution with different friction loads when *t* = 466 s: (**a**) *F* = 10 N; (**b**) *F* = 20 N; (**c**) *F* = 30 N; (**d**) *F* = 40 N; (**e**) *F* = 50 N.

The contact stress distribution of the workpiece with different friction loads is shown in Figure 11. It can be seen from the figure that the maximum contact stress always appears at the center of the wear area, and the contact pressure gradually decreases towards the surroundings with the action of different friction loads. However, the contact stress at the edge of the wear area is significantly higher than that inside, which is mainly caused by stress concentration. Figure 11 also shows that both the contact stress and the wear area of the workpiece increase as the friction load rises.

(2) The influence of friction frequency on the wear resistance of the hardened layer

**Figure 12:**
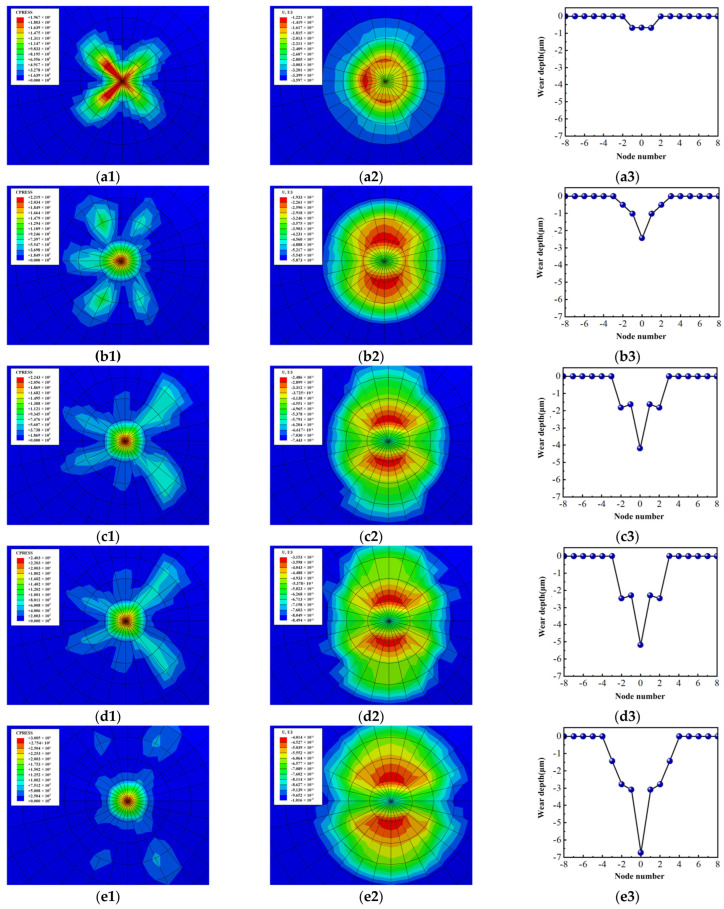
Wear process of the friction pair at different times when *f* = 2.0 Hz: (**a1**–**a3**), *t* = 66 s; (**b1**–**b3**), *t* = 266 s; (**c1**–**c3**), *t* = 467 s; (**d1**–**d3**), *t* = 665 s; (**e1**–**e3**),*t* = 865 s. (**a1**–**e1**), contact pressure of Si_3_N_4_ ball; (**a2**–**e2**), wear distribution of Si_3_N_4_ ball; (**a3**–**e3**), wear profile of hardened workpiece.

Figure 12 shows the contact pressure, wear distribution of the Si_3_N_4_ ball, and wear profile of the workpiece with the condition of *f* = 2.0 Hz at different friction times. From Figure 12(a1–c1), we can see contact between the workpiece and the Si_3_N_4_ ball at the initial stage of friction. Then, the friction pair quickly changed to surface contact when *t* = 66 s because of the relatively high friction frequency. At this moment, the maximum contact stress acts on a relatively large range on the Si_3_N_4_ ball, which results in the fact that the maximum wear area is located around its center. Therefore, a wear profile in the shape of a trapezoidal groove is formed on the workpiece. When *t* = 266 s, 467 s, 665 s, and 865 s, respectively, the variation laws of the contact stress and contact area of the friction pair have a similar trend. That is to say, the frictional behavior on the friction pair is in a quite stable stage as the friction progresses. The maximum contact stress appears at the center of the Si_3_N_4_ ball, resulting in the formation of a sharp groove at the position where the workpiece has the maximum wear depth. Moreover, the maximum contact stress decreased gradually because of the expansion in the contact area. Therefore, there emerged a development trend that the depth and width of wear improves with the increase of friction time step by step. What needs to be mentioned is that the wear depth of the hardened workpieces with the same friction time interval are 0.76 μm, 2.43 μm, 4.08 μm, 5.18 μm, and 6.94 μm, respectively. Based on this result, the increasing rate of the wear depth reaches the largest when *t* = 665 s~865 s. On the one hand, combined with Figure 12(d1,e1), it can be known that between the two moments, although the contact area of the friction pair gradually increases, the accelerated wear caused by stress concentration makes large contact stress on the Si_3_N_4_ ball moving from the friction edge to the center and finally focusing on the center of the Si_3_N_4_ ball, which leads to an improvement in the wear rate. On the other hand, though the friction is in a stable state from the experiments, the friction coefficient is relatively high compared with other moments.

**Figure 13:**
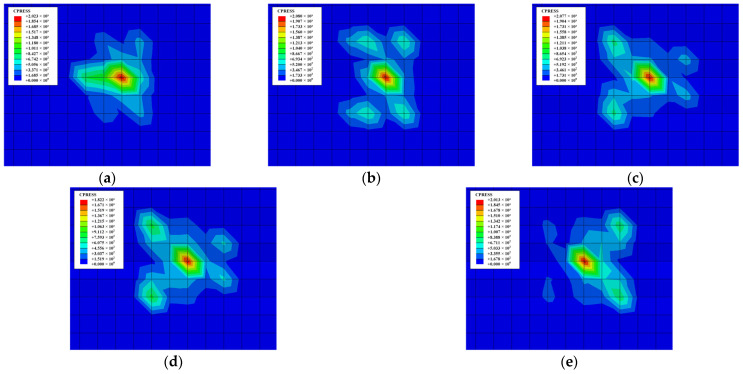
Contact stress distribution with different friction frequencies when *t* = 467 s: (**a**) *f* = 1.0 Hz; (**b**) *f* = 1.5 Hz; (**c**) *f* = 2.0 Hz; (**d**) *f* = 2.5 HZ; (**e**) *f* = 3.0 Hz.

The contact stress distribution of the hardened workpiece with different friction frequencies at *t* = 467 s is shown in Figure 13. It is found that as the frequency advances, the influence area of the contact pressure of the friction pair gradually expands by comparison. Therefore, the wear depth, width, and friction area gradually increase with the growth of the frequency. This is caused by the fact that the increased frequency makes the relative distance larger in the friction pair per unit time, so the time required to achieve the same degree of wear is shorter.

### 4.3. Validation

**Figure 14:**
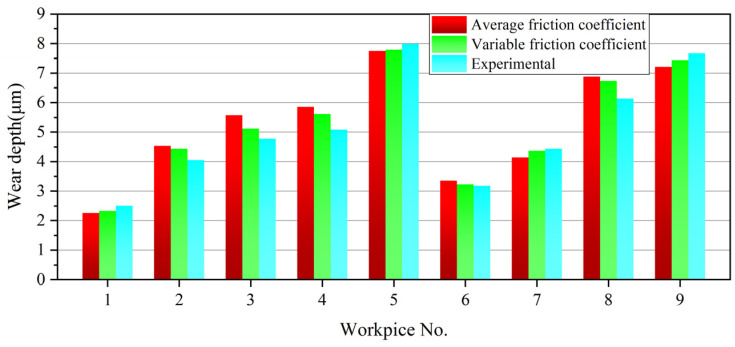
Comparison between the predictive and experimental values of the wear depth.

A comparison between the predictive wear depth and the experimental values of the hardened workpiece with different friction conditions is carried out, as shown in Figure 14. From the results, the errors between the predictive and experimental values of the wear depth with average friction coefficient and variable friction coefficient are 4.36–15.22% and 1.57–10.4%, respectively. Apparently, the prediction of the wear depth considering the variable friction coefficient is closer to the experimental results. The main reason for the error is that in the simulation, only the friction process of the friction pair under ideal conditions is studied, while the influence of the relatively high temperature on the material properties and the effect of more wear debris generated by the rough surface participating in the friction process on the wear depth are ignored. Given the small error range, it further verifies the feasibility of the prediction method for the wear depth of the hardened workpiece.

## 5. Conclusions

This paper aims to comprehensively explore the influence of friction conditions on the wear resistance of the hardened layer in the grinding process. By combining physical experiments with simulation studies, the wear process between the hardened layer and the Si_3_N_4_ ball is investigated, and the effective prediction of the wear depth is achieved.

(1) A grind-hardening experiment is carried out to discover that with certain grinding parameters (especially with a relatively large grinding depth), the initial microstructure (proeutectoid ferrite and pearlite) transforms to martensite on the workpiece surface of AISI 1405 steel. Meanwhile, because of the heat conduction inside the workpiece, the microstructure on the workpiece surface is unevenly distributed, which is divided into the complete metallurgical zone, the transition metallurgical zone, and the matrix according to the state of the microstructure distribution.

(2) An experiment on friction and wear of a hardened workpiece and Si_3_N_4_ ball with different friction conditions is conducted. Results have shown that the friction coefficient exhibits variation laws of growing first and then leveling off with the change of parameters. As the friction load goes up, the average friction coefficient gradually decreases. On the premise of an increase in friction frequency, the average friction coefficient shows a trend of first rise and then drop. Simultaneously, both the wear depth and wear rate rise with the continuous improvement in friction load and frequency.

(3) Based on physical experiments and the Archard wear model, finite element simulations are carried out for the wear process between the hardened workpieces and Si_3_N_4_ ball with different friction conditions. Results show that as the friction proceeds, the maximum contact stress always appears at the center of the contact surface of the friction pair, and the friction area gradually increases with the same friction load or frequency. Meanwhile, the depth and width of wear expand with the rise of friction load and frequency. Among them, the growth rate of the wear depth is the largest in the final stage of friction due to the influence of the change in friction coefficient.

(4) By comparison, there are small errors between the predictive values and experimental values of the wear depth of the hardened workpiece with both average friction coefficient and variable friction coefficient. Considering different friction conditions, the errors between the two values are 4.36–15.22% and 1.57–10.4%, respectively. On this account, the research on predicting the wear depth of the hardened workpiece has extremely high feasibility.

## Figures and Tables

**Table 1:** Experimental scheme for friction and wear.

No.	1	2	3	4	5	6	7	8	9
Load *F* (N)	10	20	30	40	50	50	50	50	50
Frequency *f* (Hz)	3.0	3.0	3.0	3.0	3.0	1.0	1.5	2.0	2.5
Sliding distance *s* (mm)	10								
Time *t* (min)	15								

**Table 2:** Material properties of the hardened workpiece and Si_3_N_4_ ball.

Item	Density (kg/m^3^)	Young’s Modulus (Mpa)	Poisson’s Ratio
Hardened workpiece	7.8 × 10^−9^	2 × 10^5^	0.3
Si_3_N_4_ ball	3.3 × 10^−9^	3 × 10^5^	0.26

## Data Availability

The original contributions presented in this study are included in the article. Further inquiries can be directed to the corresponding author.

## References

[1] Dong Z.G., Xu N.W., Zhang Y., Han L., Kang R.K., Wu X.F., Wang Y. (2022). Mechanism of gradient strengthening layer formation based on microstructure and microhardness of Inconel 718 grinding surface. Int. J. Adv. Manuf. Technol..

[2] Zhang Y., Fang C., Huang G., Xu X. (2018). Modeling and simulation of the distribution of undeformed chip thicknesses in surface grinding. Int. J. Mach. Tools. Manuf..

[3] Feng Z.Q., Yi H., Shu A.H., Tang L. (2024). Simulation of grinding surface topography considering wheel wear and wheel vibration. Int. J. Adv. Manuf. Technol..

[4] Yi J., Wang X.R., Song Q.H., Han D., Xiang J.F. (2024). Exploring multi-deformation mechanism and control of arc thin-walled structures during supercritical CO_2_ assisted micro milling. J. Manuf. Process.

[5] Shi X.L., Zhang X.M., Xiu S.C. (2021). A research on the mechanism and model of surface micro-damage in grinding hardening. Adv. Mech. Eng..

[6] Guo Y., Xiu S.C., Liu M.H., Shi X.L. (2017). Uniformity mechanism investigation of hardness penetration depth during grind-hardening process. Int. J. Adv. Manuf. Technol..

[7] Brinksmeier E., Brockhoff T. (1999). Surface heat treatment by using advanced grinding processes. Metall. Ital..

[8] Brockhoff T. (1999). Grind-hardening: A comprehensive view. CIRP Ann. Manuf. Technol..

[9] Zarudi I., Zhang L.C. (2002). Mechanical property improvement of quenchable steel by grinding. J. Mater. Sci..

[10] Wang G.C., Liu J.D., Pei H.J., Jia Z.H. (2006). Study on forming mechanism of surface hardening in two-pass grinding 40Cr steel. Key Eng. Mater..

[11] Nguyen T., Zarudi I., Zhang L.C. (2007). Grinding-hardening with liquid nitrogen: Mechanisms and technology. Int. J. Mach. Tools. Manuf..

[12] Liu M.H., Zhang K., Xiu S.C. (2017). Mechanism investigation of hardening layer hardness uniformity based on grind-hardening process. Int. J. Adv. Manuf. Technol..

[13] Salonitis K. (2017). A hybrid cellular automata-finite element model for the simulation of the grind-hardening process. Int. J. Adv. Manuf. Technol..

[14] Deng Y.S., Xiu S.C. (2017). Research on microstructure evolution of austenitization in grinding hardening by cellular automata simulation and experiment. Int. J. Adv. Manuf. Technol..

[15] Ehle L., Kohls E., Richter S., Spille J., Schwedt A. (2018). Grind hardening: Correlations between surface modifications and applied internal loads. Procedia CIRP.

[16] Huang X.M., Ren Y.H., Wu W., Li T. (2019). Research on grind-hardening layer and residual stresses based on variable grinding forces. Int. J. Adv. Manuf. Technol..

[17] Guo Y., Liu M.H., Yan Y.T. (2021). Hardness Prediction of Grind-Hardening Layer Based on Integrated Approach of Finite Element and Cellular Automata. Materials.

[18] Shi X.L., Xiu S.C., Liu X. (2021). Experiment study on the corrosion resistance of the surface metamorphic layer of grinding. Sci. Rep..

[19] Lerra F., Ascari A., Fortunato A. (2022). Hardness penetration depth prediction in the grind-hardening process through a combined FEM model. Procedia CIRP.

[20] Mao C., Zhang D.J., Hu Y.L., Zhang M.J., Luo Y.Q. (2024). Formation mechanisms of affected layers induced by grinding hardened AISI 52100 steel. Precis. Eng..

[21] Holmberg K., Erdemir A. (2017). Influence of tribology on global energy consumption, costs and emissions. Friction.

[22] Archard J. (1953). Contact and rubbing of flat surfaces. J. Appl. Phys..

[23] Ashish S., Kartik J., Piyush C.V. (2023). Load-dependent Finite Element Wear Simulation of Semi-Metallic and Ceramic Friction Materials Using ANSYS. Trans. Indian. Inst. Met..

[24] Li H., Ren Z., Su X., Shen L., Huang J. (2023). Study on the Fretting Wear Evolution Model of Wires with Curvature Inside Metal Rubber. Tribol. Lett..

[25] Zhang Y., Wei F., Lin S., Sun X., Liu L. (2024). Study on the Performance of Reciprocating Seals under the Coupling Effect of Elastohydrodynamic Lubrication and Rubber Wear. Eng. Res. Express.

[26] Giannopoulos G.I., Georgantzinos S.K., Anifantis N.K. (2015). Coupled vibration response of a shaft with a breathing crack. J. Sound Vib..

[27] Shu Y.J., Shen F., Ke L.L., Wang Y.S. (2022). Adaptive finite element simulation and experimental verification for fretting wear of PVDF piezoelectric thin films. Wear.

